# Virtual bedside teaching for pharmacy students during their final term at LMU Munich

**DOI:** 10.3205/zma001422

**Published:** 2021-01-28

**Authors:** Yvonne Marina Pudritz

**Affiliations:** 1 LMU Klinikum, Apotheke, Munich, Germany; 2 Ludwig-Maximilians-Universität (LMU), Department Pharmazie-Zentrum für Pharmaforschung, Munich, Germany

**Keywords:** pharmacy education, digitalisation, digital teaching, bedside teaching

## Abstract

At Ludwig-Maximilians-Universität (LMU) bedside teaching (BT) for pharmacy students has been in place since 2014. To continue offering BT during the contact restrictions imposed by the authorities in 2020, the course was digitalised, and virtual bedside teaching implemented. Using Moodle, the original concept was divided into smaller sections and presented, e.g. in the form of video sequences. All sections of the course were accessed asynchronously by the students. Tasks were individually processed and evaluated. Virtual awards were used to increase the students’ motivation. Contact with each other was possible via posting in available forums or the weekly online chat consultation. A total of 70 students successfully completed the course. The evaluation of the course was very positive, with mainly technical difficulties that were criticized. The students’ feedback will be implemented in the course concept for the winter term.

## Zusammenfassung

An der Ludwig-Maximilians-Universität (LMU) gibt es seit 2014 einen strukturierten Unterricht am Krankenbett für Pharmaziestudierende. Um diesen auch während der 2020 behördlich verordneten Kontaktbeschränkungen weiter anbieten zu können, wurde der Kurs digitalisiert und der Unterricht am virtuellen Krankenbett durchgeführt. Eingebettet in ein Moodle-Modul wurde das Präsenzkonzept in kleinere Abschnitte unterteilt und z.B. in Form von Videosequenzen dargestellt. Alle Abschnitte des Kurses wurden asynchron von den Studierenden bearbeitet. Alle Aufgaben wurden individuell bearbeitet und bewertet. Virtuelle Auszeichnungen wurden eingesetzt, um die Motivation der Studierenden zu steigern. Kontakt untereinander war möglich in Form von Forenbeiträgen oder der wöchentlichen online Sprechstunde. Insgesamt konnten 70 Studierende den Kurs erfolgreich beenden. Evaluation des Angebotes war sehr positiv, bemängelt wurden hauptsächlich technische Schwierigkeiten. Das Feedback der Studierenden wird für das Wintersemester in das Kurskonzept implementiert.

## 1. Introduction

In medicine, bedside teaching (BT) is an important part of university education. BT is considered a fundamental method for learning clinical and communication skills [1]. Clinical pharmacy is a relatively new subject in the otherwise very scientific curriculum of pharmacy. It requires more than the usual acquisition of knowledge to transfer theory into clinical practice. While this should be predictable and calculable in a chemical experiment, many other factors, which cannot necessarily be controlled, are added in clinical pharmacy. Contrary to medical education, BT is the exception rather than the norm for pharmacy students. At the Ludwig-Maximilians-Universität (LMU), visits to hospitals by pharmacy students have been taking place since 2005. Since 2014, these visits have been supplemented by a standardized concept for BT in small groups [2]. To provide BT for pharmacy students even during existing contact restrictions, the teaching concept for the seminar Clinical Pharmacy and practical bedside teaching has been digitalised in a Moodle module.

## 2. Project description

### 2.1. Setting

The virtual BT takes place in the 8^th^ term of Pharmacy studies as a Moodle course.

#### 2.2. Concept & structure

The concept for digitalisation is based on Hege's aspects of implementing e-learning [3]. The original BT (face-to-face) is already organized via Moodle [https://moodle.de/], the existing module was expanded for digital teaching. The normal day at the clinic (4-5h) was divided into smaller sections and made available asynchronously. This allowed all students to conduct the course in their own pace, but within a given time frame. Thus, all students had to determine the appropriate working schedule for themselves. Since the hospital’s own network could not be used, the students were provided with corresponding but freely available web links and literature sources to use from home. Each student worked individually; exchange was possible via various forums in Moodle. Table 1 (Tab. 1) shows the comparison between normal and virtual BT. By tracking the completion of individual activities, both students and lecturers maintained an overview of work completed or still to be completed. Feedback was provided in the form of a weekly online chat, various forums and on completed tasks. To increase the intrinsic motivation of the students, virtual badges were used [4], [5].

#### 2.3. Implementation

The virtual BT was held for the first time during the summer term 2020. The course took place from May 8^th^ to June 5^th^. A total of five tasks had to be completed at regular intervals. Seven badges with different degrees of difficulty were defined, the conditions for receiving each badge were openly visible to all students.

#### 2.4. Evaluation

An evaluation of the Moodle module was performed as usual for the face-to-face BT with Evasys^®^ [http://www.evasys.de].

## 3. Preliminary results

There were 72 students enrolled in total, 70 successfully completed the course. Of the seven badges, five were awarded, but no one had fulfilled the conditions for the two most time-consuming ones by the end of term. Eight students started to work on additional material provided for this purpose. The general course evaluation was completed by 41% of the students (n=29). Of these, the majority (86%) gave the course the grade “very good/good”. The students liked the independent, individual work and the personal feedback. They spent more time on the homework than indicated prior to the assignment, and there were also several technical difficulties, e.g. opening or filling in documents. An overview of the entire course at the beginning of the module was also lacking.

## 4. Discussion

Feedback on the virtual BT was very positive. It may have been helpful that the period of the course coincided with the strict initial restrictions in Bavaria and that the students did not have to attend any other classes or courses at the same time. This may have led to more intensive research, which in turn led to the criticized longer time spent on assignments. From the individual feedback on the assignments it became apparent that the instructions for the tasks need to be more focussed so that students avoid unnecessary work. Many of the technical problems were solved during the course, partly in cooperation with the students. Discussions with the LMU Moodle team are planned for the remaining problems. The students liked the asynchronous structure as well as the individual feedback on the given tasks. The students' motivation for exchange seemed to be higher than during the original face-to-face event. There was more visible interaction among the students in comparison during summer. The tracking of progress through activity completion during the course was mainly used by the lecturers and was not seen as helpful for course organization by all students. For the next term, an overview of the entire process will be provided from the start.

## 5. Conclusion

Teaching at a virtual hospital bed does not replace contact with real patients, but it could be easily accommodated in the curriculum and currently offers an adequate substitute. For the winter term, a further expansion based on previous students’ feedback is planned. Further video sequences are planned to make the patients appear more “real”, as well as a graphic semester overview to support self-organization. The e-learning elements will continue to be used when face-to-face classes resume, in order to prepare the students for the BT with real patients.

## Competing interests

The author declares that she has no competing interests.

## Figures and Tables

**Table 1:**
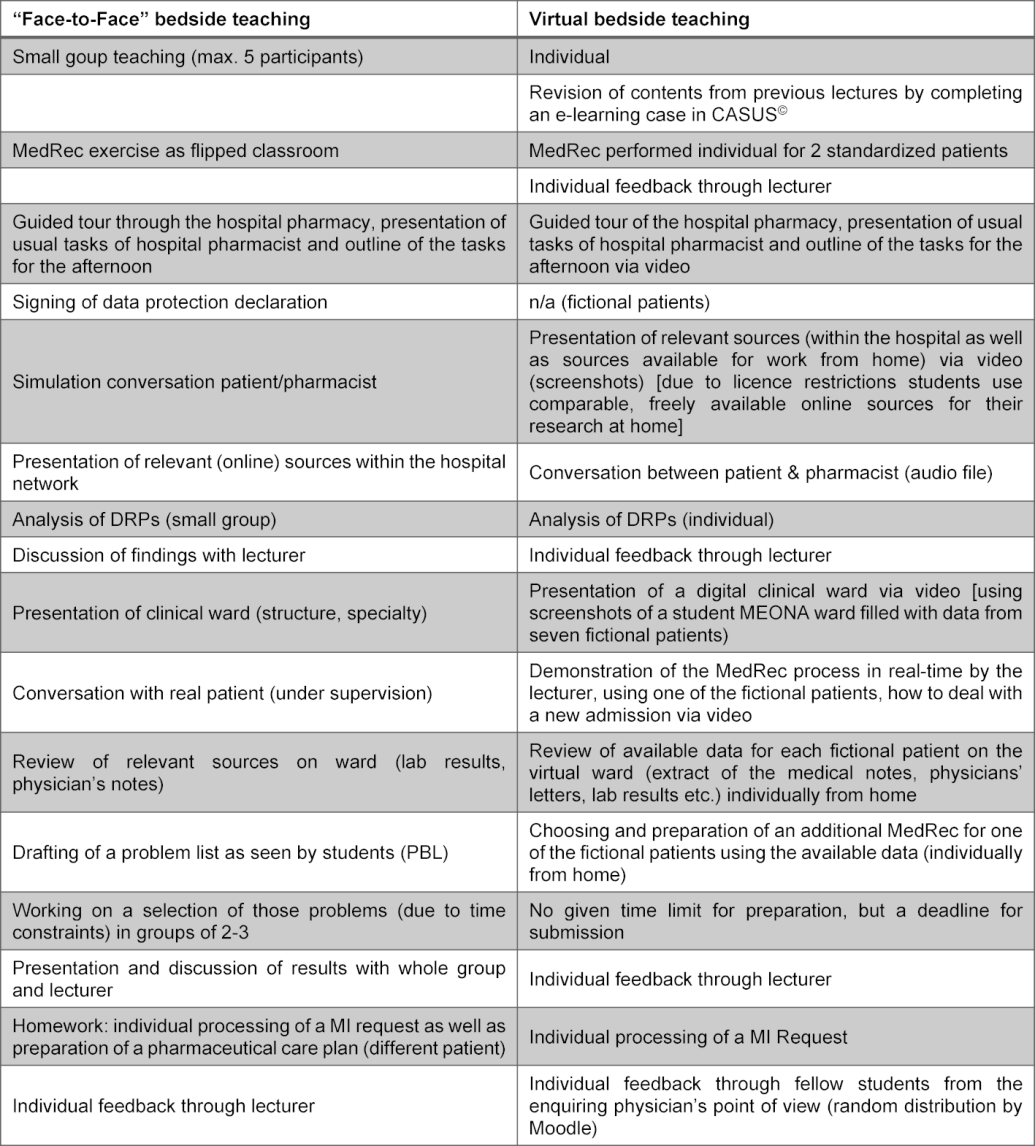
Comparison of the schedules for the original bedside teaching (“face-to-face”) with the virtual version. Abbreviations used: DRP – drug related problem, MedRec – medicine reconciliation, MEONA – electronic prescribing system, MI – medicines information, PBL – problem based learning.

## References

[1] Peters M, ten Cate O (2014). Bedside teaching in medical education: A literature review. Perspect Med Educ.

[2] Pudritz YM, Wahl-Schott C, Noller J, Beitz-Radzio C, Kugelmann D, Sontheimer S, Westernholz S (2019). Neue und moderne didaktische Methoden in der Klinischen Pharmazie. Methoden in der Hochschullehre. Interdisziplinäre Perspektiven aus der Praxis (Perspektiven der Hochschullehre)..

[3] Hege I (2020). Kurze Zusammenfassung von Aspekten, die bei der Umsetzung von E-Learning wichtig sind.

[4] Sailer M, Hense JU, Mayr SK, Mandl H (2017). How gamification motivates: An experimental study of the effects of specific game design elements on psychological need satisfaction. Com Human Behav.

[5] Tolks D, Lampert C, Dadaczynski K, Maslon E, Paulus P, Sailer M (2020). Spielerische Ansätze in Prävention und Gesundheitsförderung: Serious Games und Gamification. Bundesgesundheitsbl.

